# Prospective Randomized Observational Pilot Trial Evaluating the Effect of Different Durations of Interdisciplinary Early Intervention and Family Support in Parents of Very Low Birth Weight Infants (Early Bird Study)

**DOI:** 10.3389/fpubh.2020.00242

**Published:** 2020-07-03

**Authors:** Bernhard Resch, Claudia Hofbauer-Krug, Jasmin Pansy, Karin Prechtl, Alexander Avian, Ronald Kurz

**Affiliations:** ^1^University Course for Interdisciplinary Early Intervention and Family Support, Postgraduate School, Medical University of Graz, Graz, Austria; ^2^Research Unit for Neonatal Infectious Diseases and Epidemiology, Medical University of Graz, Graz, Austria; ^3^Division of Neonatology, Department of Pediatrics and Adolescent Medicine, Medical University of Graz, Graz, Austria; ^4^Institute for Social and Orthopedagogic Interventions (SHFI), Graz, Austria; ^5^Institute for Statistics, Medical University of Graz, Graz, Austria

**Keywords:** interdisciplinary early intervention, family support, preterm infant, neonatal intensive care unit, duration, questionnaire, Likert scales, very low birth weight

## Abstract

**Background:** Early childhood intervention (ECI) is a holistic approach for infants with or at risk for psychomotor and/or cognitive and/or behavioral impairment. It aims to optimally support them and positively influence their neurodevelopmental outcome. The right dosage of intervention and when the intervention should start are still to be determined. Hypothesis: Parents are more satisfied when the duration of ECI is longer (120 min once a week) than the usual 90-min session.

**Methods:** We developed a parental questionnaire (both mother and father) that evaluated the level of satisfaction of parents with the intervention. We compared 120 with 90 min of ECI per week during the school year 2017/18. Included were parents of very low birth weight infants (<1,500 g) following informed consent. ECI was initiated at the NICU at an infant age of ≥ 2 weeks. Parents were randomized (https://www.randomizer.at/) to a 120- or 90-min duration and had to answer the questionnaire to the approximate time-point of 1, 3, and 6 months. Answers were classified as strongly agree, agree, neither agree nor disagree, disagree, and strongly disagree except for the last question, which directly rated the ECI professional.

**Results:** Eleven fathers (55%) and 19 mothers (95%) of the 10 parents of each group participated in the study. Demographic data did not differ between groups, and the median time-points of questionnaire answers were 77, 137, and 220 days, respectively. Overall, 120-min ECI sessions were not superior to 90-min sessions for both parents regarding parental satisfaction during the study time. We found no differences between fathers and mothers and minimal changes over time. All parents were satisfied with the ECI professionals, irrespective of ECI duration.

**Conclusion:** An ECI duration of 120 min once per week was not superior to a 9- min duration regarding parental satisfaction with ECI professionals and their work.

## Introduction

Very low birth weight (VLBW) infants remain a challenge for neonatologists and neonatal nurses, and very preterm birth is a well-recognized risk factor for motor, cognitive, and behavioral impairment in the developing child (1, 2). Early intervention utilizes the relatively high plasticity of the developing brain in early childhood, which is observed from about 2 months before term to about 6 months after term age (3). Parents are faced with numerous concerns, fears, and uncertainties regarding their preterm babies. Mothers of very preterm infants experience a high level of stress for many reasons, particularly regarding their child's medical condition and well-being, and the influence of development, temperament, and maternal depression on parenting stress levels is well-known (4). Parents worry about future psychomotor and somatic development and thus ask for early support and intervention (5–8). Interestingly, nurture-based interventions in the NICU have been demonstrated to positively influence the early mother–infant relationship in ways that have long-lasting effects on the developmental trajectory of the brain and behavior (9).

The limitations of studies and particularly randomized controlled trials on the topic of early childhood intervention (ECI) include the ethical dilemma regarding the control group (1). If the controls receive the same or a comparable intervention, the differences will be small and may not be measurable, but otherwise, the controls would seem to be severely neglected. Also, is the developmental quotient the right end-point of studies—what about other disturbances? Further open questions are, for example, when the intervention ideally should start (the earlier, the better?), as effects flatten over the years, and how much intervention (right dosage) would be optimal for the child and the family (1, 10).

ECI is generally developed to improve child outcomes by changing the behavior of early childhood educators or parents (11). The dosage has to be considered firstly at the ECI level, meaning how much time the child will be learning for or how much time the ECI professionals need for activity implementation, and secondly at the parents' and child's level, meaning the amount of intervention that is provided to children or to the adults who care for them. Additionally, there may be differences between the “dosage intended” (the 90-min unit for a home visit) and the dosage offered (that which the ECI professional provides to parents or the child). Ultimately, there will be a certain amount of “dosage received,” which might vary for any reason, e.g., only 9 instead of 12 visits in case of hospitalization of the child for 2 weeks (11).

There is no clear definition of what is meant by “early” and no evidence favoring, for instance, initiation of intervention programs at 3 months of age as compared with 12 months (10). Early intervention might be defined as multidisciplinary offers for children up to the age of 5 years that promote health and well-being, affirm necessary competences, minimize developmental delays, prevent or positively influence impairments, and functional deteriorations, and finally support parenthood and family functioning in general (12). The rationale for the study was to initiate a discussion on the fact that ECI duration had been constituted to be 90 min once per week, and some ECI professionals felt that this might be insufficient.

Our hypothesis was that parents of VLBW infants would be more satisfied when the duration of ECI is longer (120 min) than the usual 90 min once per week.

## Materials and Methods

In a prospective randomized pilot study, we investigated, by use of a self-developed questionnaire, whether parents of VLBW infants are more satisfied with ECI units of 120 min compared to 90 min once per week of standardized ECI as described elsewhere (13, 14).

**Figure 1:**
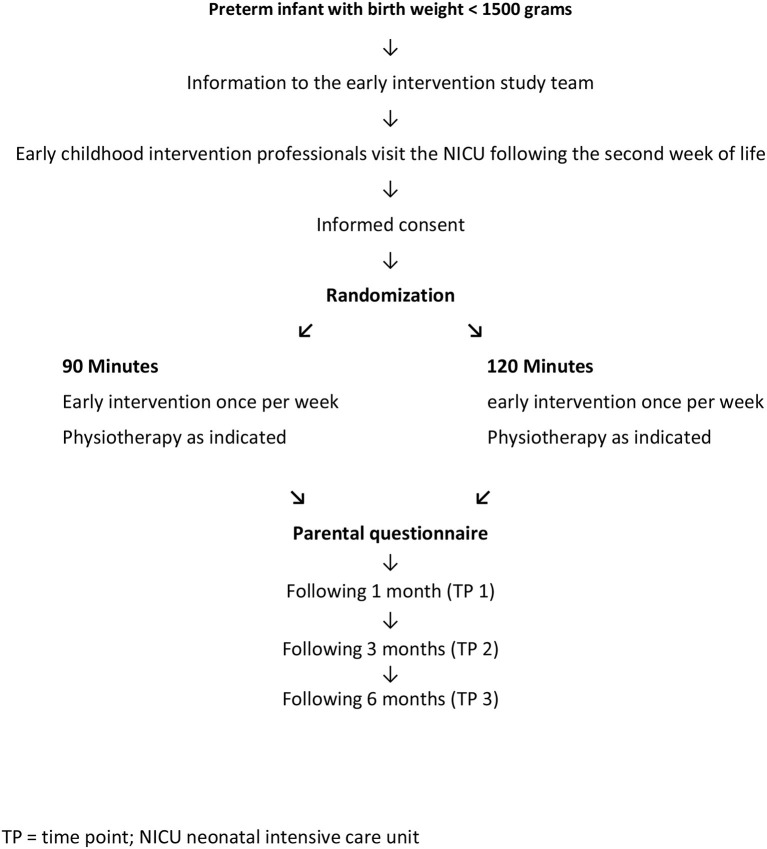
Study design of the prospective observational testing of two different regimens of either 90- or 120-min early intervention in the families of very low birth weight infants.

Parents of a preterm infant with a birth weight below 1,500 g were consecutively asked to participate in the study during the winter of 2017/18 until May 2018. Eligibility criteria applied to a total of 83 out of 105 parents of preterm infants below 1,500 g hospitalized at our NICU during 2017 and 2018. Mismatches were language barriers in 21 parents and having no husband for one mother. Following informed consent, parents were randomized (https://www.randomizer.at/) by the statistician (A.A.) to the two different ECI durations (units of either 120 or 90 min), which started at the neonatal intensive care unit (NICU 2) of the Division of Neonatology of the Medical University of Graz (see flow chart in Figure 1). The study was approved by the local ethics committee (EK 29–147 ex 16/17) and started in winter 2017/18.

The questionnaire was developed by the ECI professionals, was validated by volunteers, and was thereafter rephrased or partly deleted by a psychologist experienced in setting up questionnaires. Questions had to be answered by the father and mother at 1, 3, and 6 months of age of the infant (time points 1, 2, and 3). The answers were built according to Likert scales, and the standard answers (strongly disagree; disagree; neither agree nor disagree; agree; strongly agree) were slightly modified through translation to German, but the meaning was essentially the same (15). These answers are typical for a pilot trial using a lot of items, as was done in our study (14). The questionnaire included 29 questions that could be answered by use of 1 of the 5 categories, but the last question, number 30, could be answered using “very good, good, neither good nor poor, poor, very poor.” In a supplementary file (Supplementary Data Sheet 1), we include the original German questionnaire and the data analysis by the statistician (A.A.). Other questions included the age of the parent and the birth weight and gender of the infant. The questionnaire is presented in Table 1. In total, only 11 fathers but 19 mothers answered the questionnaire at all three time points. For many years, our parents have been seen by clinical psychologists. Of these, one psychologist only works at the NICU. Additionally, Christian chaplains are available at 24 hours a day.

**Table 1:** Questions of the self-developed questionnaire used for study purposes.

1	Does the ECI professional take you and your situation seriously?
2	Do you understand the explanations of the ECI professional?
3	Were the talks with the ECI professional too short?
4	Were the talks with your ECI professional too rare?
5	Was the ECI professional responsive to your wishes?
6	Was the ECI professional motivated?
7	Did you look forward to the regular visits of your ECI professional?
8	Did the ECI professional provide you with more security in handling your child?
9	Could the ECI professional reduce your fears regarding the future of your baby?
10	Do you feel encouraged by your ECI professional regarding the future of your baby?
11	Do you have more time for your own needs due to the support of your ECI professional?
12	Do you meet friends regularly?
13	Does the ECI professional support doctor and administrative visits at public offices?
14	Do you discuss partnership problems with the ECI professional?
15	Do you feel comfortable during your daily routine with your child?
16	Is the ECI professional responsive to the characteristics of your infant?
17	Does the ECI professional give you suggestions about how to work with your child?
18	Is the ECI professional friendly?
19	Does the ECI professional include other family members?
20	Does the ECI professional inform you on further interventions for your child?
21	Is the ECI professional on time for fixed appointments?
22	Is the expert co-working with a physiotherapist?
23	Do you sleep well?
24	Does the expert regularly examine neuro-motoric and cognitive development?
25	Do you recommend the ECI professionals for comparable reasons?
26	Can you imagine retrying ECI for your next child?
27	Do you have enough time for yourself?
28	Do you have someone to talk about different problems?
29	Do you feel healthy?
30	How do you rate the work of the ECI professional?

*ECI, early childhood intervention*.

## Descriptive Analysis and Statistics

All data were collected by the statistician. Answers were given in color tables (descriptive interpretation) and column diagrams (Supplementary Data Sheet 1, page 3). The figures in Supplementary Data Sheet 1, part 1.1 show results for all 11 fathers and 19 mothers. Row 1 depicts the three time points (roughly at 1, 3, and 6 months of age of the baby), while left-sided columns belong to the fathers and right-sided columns to the mothers. Six fathers and 10 mothers were randomized to 120-min units. The remaining five fathers and nine mothers were in the 90-min unit group. The same questionnaire was used at the three time points, at 1, 3, and 6 months. The figures in part 1.2.1 (Supplementary Data Sheet 1, page 11) show 3 condensed columns representing the combined answers “strongly agree” and “agree,” the single answer “neither agree nor disagree,” and the combined answers “disagree” and “strongly disagree” for both fathers and mothers at time point 3 (~6 months). For numerical data, a *t*-test and, for categorical data, Fisher's Exact test were used regarding perinatal parameters. The significance level was set at < 0.05.

## Results

According to the perinatal data shown in Table 2, there were no differences between groups. The results of the questionnaire are given in a descriptive summary. Eleven fathers (55%) and 19 mothers (95%) participated in the questionnaire rounds during the study period.

**Table 2:** Perinatal parameters of the study population.

**Parameter**	**Total**	**120 Min**	**90 Min**	***p*-value**
Maternal age (years)	29 (27–36)	31 (28–34)	29 (27–36)	0.952^*^
Paternal age (years)	34 (29–36)	35 (30–35)	30 (29–36)	0.733^*^
Time point 1 (days)	77 (59–90)	68 (62–83)	89 (59–146)	0.325^*^
Time point 2 (days)	137 (123–179)	127 (123–142)	157 (126–209)	0.108^*^
Time point 3 (days)	220 (210–234)	219 (211–234)	226 (209–268)	0.811^*^
Birth weight (grams)	1,060 (770–1,120)	1,060 (860–1,080)	1,068 (730–1,198)	0.78^*^
Gender female	7 (35)	5 (50)	2 (20)	0.35^**^
Gender male	13 (65)	5 (50)	8 (80)	

*Data are given as median (interquartile range 25–75) or n (%)*.

* *t-test*;

** *fisher exact test*.

Questions 1–4: Talking with the ECI professionals was well-recognized, but parents did not feel that having more time for discussion during the 120-min units would be useful. In contrast, the longer units were more often thought to be not too rare.

Questions 5–8: The motivation of the ECI professionals was always affirmed by the parents, as was the ECI professional's fulfillment of certain parental wishes. This fact did not change during the study. The first 120-min unit seemed to be exhausting for both father and mother. This was not the fact at the next time points. The longer units did not result in higher rates of parents feeling safe during routine care of the infant.

Questions 9–12: Anxiety and reinforcement were the same for both units. The questions on “more time for personal duties” or “meeting friends” could not be clearly answered, and there was no trend during the study period. Thus, these questions remained more or less a problem for the parents, independent of the duration of the ECI unit.

Questions 13–16: Support regarding doctors' visits/administrative procedures was more positively described by the 90-min unit group. The majority of parents did not have discussions of partnership problems with the ECI professional. A minority of mothers did discuss partnership problems with the ECI professional. Parents felt comfortable with their babies regardless of the duration of the units and appreciated the professionals' handling of their infants and children.

Questions 17–20: Co-working with other family members was a difficult task that did not improve during the longer units. Information provided by the ECI professional was well-accepted by all parents during the study. The ECI professionals were always classified as being friendly, and their help regarding parental education and training was well-recognized, with improvements during the study.

Questions 21, 22, and 24: ECI professionals were very well-organized and always arrived on time. They worked well with physiotherapists. In the view of the parents, this co-working was more effective in the shorter ECI units. Testing and judging of the infants' development by the ECI professionals were finally recognized at the end of the study.

Questions 23, 27–29: Parents slept well (and better when their child had shorter ECI units), and “enough time for each other” was answered positively. Discussing problems with the ECI professional was done by several mothers at the end of the study period (no father did so).

Questions 24, 25, and 30: If the extreme situation of having had a preterm infant in an NICU were to occur again, the parents indicated that they would to try to get support from ECI professionals. All parents would again recommend ECI professionals to parents living in a comparable situation. Finally, the ECI professionals were all judged as having been very good.

The most divergent answers concerned the following problems: the parent felt that she/he did not have enough time for herself/himself; difficulties in having time to visit friends regularly; support for doctors' visits; having someone to talk to about problems.

## Discussion

The main finding of our pilot trial was that the usual sessions of 90 min ECI once per week were not inferior to 120 min once per week for both mother and father over a 6-month observation period. This was surprising for the study team and disproved our hypothesis. Hence, the important thing when a session duration is given as minutes per ECI visit is the threshold, that is, the specific dosage level at which an intervention affects outcome. Hence “dosage is not a one-size-fits-all concept” (11). This raises some questions, including the following. Does the amount of the intervention directly affect the size of expected outcomes? Does dosage matter for a full session or for specific strategies used within that session? (11) And besides organizing activities, questions should be addressed regarding, “Are there enough staff available and how should absences of the parents/children be dealt with?” (11). ECI is a comprehensive service provided for children with developmental problems or for those at a high risk of having developmental problems and thus implies the active participation of families in the intervention process (16). The family is less part of the intervention objective than rather a resource, in so far that the family is the expert on its children making all necessary decisions according to the children's needs and being attended to by the ECI professionals (17, 18). ECI consists of multidisciplinary services to promote child health and well-being, enhance emerging competencies, minimize developmental delays, remediate existing or emerging disabilities, prevent functional deterioration, and promote adaptive parenting and overall family functioning (19, 20).

There was no doubt, considering the answers to questions 24, 25, and 30, that the ECI professionals did a good job and that parents were extremely satisfied. The recommendations of the ECI professional are known to be sometimes complex, confusing, or even contradictory for parents. Thus, organization of the visits of the professionals plays a key role and is often an overwhelming task, even for very conscientious parents (17). Looking at the answers to question 21, this was no problem during the study time. In the literature, service coordination is still found to be a major challenge in the ECI field (21). Hadders-Algra summarized the effectivity of early intervention as follows (22): “(1) coaching of parents seems an effective means of intervention; (2) our understanding of the plasticity of the developing human brain is currently too limited to allow a direct practical implementation in early intervention; (3) intervention before term age should primarily focus on stress reduction, intervention after term age on stimulation of infant development; and (4) our knowledge of the best ways to stimulate infant development is scant” (22). The reduced stress level of the parents was positively recognized by overall positive answers to question 7 (the regular weekly sessions with the ECI professional was well-appreciated, with solely positive answers at 6 months).

Problems selected out of the most divergent answers included social phenomena that we interpreted as being mainly not associated with the ECI professional but more related to having a disabled child, the care of whom might be time-consuming (problems with meeting friends, having someone to talk to about problems, getting support at doctors' visits, and having time for themselves).

Discussion of the key components of early developmental interventions has revealed that the intervention should be initiated as early as possible and ideally in the NICU (23). This was our approach too, and it was successfully and simply introduced at our NICU. In interviews with the ECI professionals after our pilot trial had finished, they all reported that they had been happy when starting the ECI study program at the NICU despite a high degree of respect for and fear regarding neonatal intensive care. Parents and the home environment have the strongest, most enduring influence on child development irrespective of socio-economic status and level of parental education (23). Given the critical role that parents have for ECI with preterm infants, it is important to consider factors that may negatively influence parental functioning and ability to engage with ECI for their child. The main such factors are parental mental health problems, including anxiety and depression (10, 22, 23). Heterogeneity between interventions (timing, focus, and length) increases the challenges to parental mental health (23).

Even for children presenting with highly complex and unusual developmental and behavioral patterns, building relationships with both parents and therapists has been a central theme (17). Questions 10, 14, 16, 18, and 19, for example, consider this part of the ECI program, and, of course, question 22 is important for to obtaining information regarding the interdisciplinary approach of the ECI professional.

Bagnato et al. (24) proposed a definition of “dosage” in early intervention services as the “amount of time that an individual child must engage and participate in an ECI program or service to show measurable functional progress.” This definition recognizes that “dosage” captures more than just number of hours or days of service provided (24). This dosage-finding is far beyond the objectives of our study. We can only answer the question of whether 120-min sessions are better-tolerated or as tolerated as 90-min sessions by the parents receiving weekly sessions in our county. In the words of Jung (25): “We continue to choose “1x/week” because that's the trend; it's just the way we've always done it. With the push in our field to implement evidence-based practices, it's a real challenge that we don't actually have evidence for this important decision” (25).

A review including 14 studies on current practices in the management of parental satisfaction with ECI reported on a key element that remained unanswered to a certain degree because information on measurement tools used was missing and information on the reliability and validity of the measurement instruments was often unavailable (26). These findings were the spur to our study—the creation of this German questionnaire and the pilot trial on parental satisfaction with different ECI durations once per week.

Two recent reviews (22, 23) focused on infants at risk for or with definite diagnosis of cerebral palsy. Hadders-Algra et al. (22) analyzed seven studies in detail, and Morgan et al. (23) included 34 studies in their systematic review. They both found the dosing of the intervention to be crucial for the success or failure of the inventions (22, 23). The problems mentioned in both reviews included the heterogeneity of the studies concerning the method and duration of interventions. Multifaceted intervention might offer the best opportunities for both child and parents (22, 23). A Cochrane review of early intervention programs for preterm infants concluded that early intervention programs focusing on the parent–child relationship were more effective than programs focusing on the child or the parents alone (27).

A very recent narrative review on early childhood intervention in middle- and low-income countries summarized that in high-income countries, ECI is recommended for high-risk infants starting in the neonatal period and specialized interventions for children with developmental disabilities as early as 3 months of age but that less information is available regarding the timing of IEI-FS in middle- and low-income countries (28). Furthermore, emerging evidence supports the efficacy of community-based ECI that focuses on peer support, responsive caregiving, and the prevention of secondary morbidities. A combination of individual home visits and community-based groups might be the best strategy for the delivery of ECI given that scenario (28).

Before 5 months' corrected age, the most predictive tools for detecting developmental risks at term age were found to be magnetic resonance imaging, the Prechtl Qualitative Assessment of General Movements with the highest sensitivity (98%), and the Hammersmith Infant Neurological Examination, as concluded by the authors of a systematic review including six high-quality systematic reviews and two evidence-based clinical guidelines (29). Hence, the authors advised clinicians to better understand the importance of prompt referral to diagnosis-specific ECI in order to optimize the infant's motor and cognitive plasticity, prevent secondary complications, and enhance caregiver well-being (29).

A principle dilemma of ECI programs is shown in a very recent systematic review on movement-based interventions (30). Although movement-based interventions showed potential for improving body structure and function and activity outcomes for children with motor impairment, results were mostly not significant, and the main problems included small sample sizes, variable study quality, and high risk of bias (30).

The limitations of our pilot trial include the low number of fathers participating in the questionnaire rounds and the difficulties concerning interpretation of findings (color tables), resulting in very cautious wordings and avoiding stringent answers to some questions. Reflecting on monitoring, evaluation, and learning (MEL) might lead to several additional biases, as will be elucidated briefly. We tend to search for, notice, and interpret information in a way that confirms our existing views or beliefs—this might be the case regarding interpretation of more divergent answers. Again, another factor influencing MEL might be group reinforcement, meaning that we all self-censor ourselves during discussion of study findings. At least, we might have interpreted particular answers as correlated to the different durations of ECI when they were not; this is closely linked to “need for coherence,” which predisposes us to establish causal relationships when they may be non-existent (31). The measurement of change (120 vs. 90 min per week, satisfaction of parents expressed by answers to a questionnaire) does not really reflect the true complexity of the process of change. In contrast to popular belief, process performance has the biggest impact on organizational and business success and not people performance (e.g., performance improvements, employee feedback, or communication effectiveness) (32).

The strengths of the trial were the intense family work during the first 6 months of life with parents of VLBW infants, with almost no ECI sessions missed, and the relatively clear answer regarding our hypothesis. Additionally, some more divergent answers elucidated the in-family problems of parents of VLBW infants.

In conclusion, we found that 90-min weekly sessions of ECI were not inferior to 120-min weekly sessions during the 6-month study period. ECI professionals were well-respected and were judged positively overall.

## Data Availability Statement

The datasets generated for this study are available on request to the corresponding author.

## Ethics Statement

The studies involving human participants were reviewed and approved by Ethic Committee of the Medical University of Graz. The patients/participants provided their written informed consent to participate in this study.

## Author Contributions

BR was responsible for study protocol and writing the manuscript. CH-K and KP developed the questionaire and were responsible for study design and execution. JP contributed to the study design and revised the manuscript. AA was responsible for statistics. RK supervised the study and revised the manuscript. All authors contributed to the article and approved the submitted version.

## Conflict of Interest

The authors declare that the research was conducted in the absence of any commercial or financial relationships that could be construed as a potential conflict of interest.
