# Supplementary material for: Prospective Randomized Observational Pilot Trial Evaluating the Effect of Different Durations of Interdisciplinary Early Intervention and Family Support in Parents of Very Low Birth Weight Infants (Early Bird Study)
Source: Front Public Health. 2020 Jul 3;8:242. doi: 10.3389/fpubh.2020.00242 (PMC7349967; doi:10.3389/fpubh.2020.00242)
Supplement: Supplementary file 1 [file Data_Sheet_1.PDF]

Early Bird

04.12.2018

Institut für Medizinische Informatik, Statistik und Dokumentation
Medizinische Universität Graz
Auenbrugger Platz 2, 8036 Graz, Austria

PD Mag. Dr. Alexander Avian
+43-316-385-17873
alexander.avian@medunigraz.at

Inhaltsverzeichnis

1 Datensichtung 3
1.1 Antworten in allen fünf Kategorien 3
1.2 Antworten in drei Kategorien zusammengefasst 8
1.2.1 Häufigkeitstabelle der Antworten 8
1.2.2 Balkendiagramme der Häufigkeit der Antworten bei der letzten Befragung 11

Institut für Medizinische Informatik, Statistik und Dokumentation
Medizinische Universität Graz
Auenbrugger Platz 2, 8036 Graz, Austria
PD Mag. Dr. Alexander Avian
+43-316-385-17873
alexander.avian@medunigraz.at

1 Datensichtung

		Alle	Gruppe	
			120 Minuten	90 Minuten
Alter_Mutter [Jahre; Median IQR]		29 (27 – 36)	31 (28- 34)	29 (27- 36)
Alter_Vater [Jahre; Median IQR]		34 (29 – 36)	35 (30- 35)	30 (29-36)
Alter_Kind_MSG1 [Tage; Median IQR]		76,5 (59 – 90)	68 (62- 83)	88,5 (59-146)
Alter_Kind_MSG2 [Tage; Median IQR]		137 (210 – 234)	127 (123-142)	156,5 (126-209)
Alter_Kind_MSG3 [Tage; Median IQR]		220 (210 – 234)	218,5 (211- 234	225,5 (209-268)
Geburtsgewicht [g]		1060 (770 – 1120)	1060 860- 1080)	1068 (730-1198)
Geschlecht	0	7	5	2
	1	13	5	8

1.1 Antworten in allen fünf Kategorien

	Antwortender Elternteil	Vater						Mutter					
	Zeitpunkt	MZP 1		MZP 2		MZP 3		MZP 1		MZP 2		MZP 3	
	Gruppe	120	90	120	90	120	90	120	90	120	90	120	90
ernst_nehmen	ja	6	5	6	6	6	7	10	9	10	9	10	9
	eher ja	0	0	0	0	0	0	0	0	0	0	0	0
	teils-teils	0	0	0	0	0	0	0	0	0	0	0	0
	eher nein	0	0	0	0	0	0	0	0	0	0	0	0
	nein	0	0	0	0	0	0	0	0	0	0	0	0
Erklärungen_verstanden	ja	6	4	4	6	6	7	10	9	10	9	10	9
	eher ja	0	1	2	0	0	0	0	0	0	0	0	0
	teils-teils	0	0	0	0	0	0	0	0	0	0	0	0
	eher nein	0	0	0	0	0	0	0	0	0	0	0	0
	nein	0	0	0	0	0	0	0	0	0	0	0	0
Gespräche_zu_kurz	ja	0	0	1	1	1	1	0	0	1	1	1	1
	eher ja	0	0	0	1	0	0	0	0	0	1	0	1
	teils-teils	0	2	0	0	1	0	0	2	1	1	2	1
	eher nein	0	1	1	1	1	3	0	0	0	1	0	1
	nein	6	2	4	3	3	3	10	7	8	5	7	5
Gespräche_zu_selten	ja	0	0	0	0	1	1	0	0	0	0	1	1
	eher ja	0	1	0	0	0	0	0	1	0	0	0	0
	teils-teils	0	0	0	0	0	0	0	0	1	0	1	0
	eher nein	2	2	2	2	1	2	0	1	0	1	2	1
	nein	4	2	4	4	4	4	10	7	9	8	6	7

PD Mag. Dr. Alexander Avian
+43-316-385-17873
alexander.avian@medunigraz.at

	Antwortender Elternteil	Vater						Mutter					
	Zeitpunkt	MZP 1		MZP 2		MZP 3		MZP 1		MZP 2		MZP 3	
	Gruppe	120	90	120	90	120	90	120	90	120	90	120	90
Wünsche	ja	6	3	6	6	6	6	9	9	9	8	10	9
	eher ja	0	2	0	0	0	1	1	0	1	0	0	0
	teils-teils	0	0	0	0	0	0	0	0	0	1	0	0
	eher nein	0	0	0	0	0	0	0	0	0	0	0	0
	nein	0	0	0	0	0	0	0	0	0	0	0	0
motiviert	ja	5	5	6	6	6	7	9	9	10	9	10	9
	eher ja	1	0	0	0	0	0	1	0	0	0	0	0
	teils-teils	0	0	0	0	0	0	0	0	0	0	0	0
	eher nein	0	0	0	0	0	0	0	0	0	0	0	0
	nein	0	0	0	0	0	0	0	0	0	0	0	0
Freuen	ja	4	3	5	6	6	6	8	9	9	9	10	9
	eher ja	1	2	1	0	0	1	1	0	1	0	0	0
	teils-teils	1	0	0	0	0	0	1	0	0	0	0	0
	eher nein	0	0	0	0	0	0	0	0	0	0	0	0
	nein	0	0	0	0	0	0	0	0	0	0	0	0
Sicherheit	ja	3	2	2	5	4	6	3	5	8	7	6	8
	eher ja	2	2	4	1	2	1	5	2	1	2	4	1
	teils-teils	1	1	0	0	0	0	1	1	1	0	0	0
	eher nein	0	0	0	0	0	0	0	0	0	0	0	0
	nein	0	0	0	0	0	0	1	1	0	0	0	0
Ängste_vermindern	ja	3	2	2	5	2	4	4	4	4	7	3	7
	eher ja	1	2	0	1	0	2	3	3	2	2	6	2
	teils-teils	2	1	4	0	4	1	3	1	4	0	1	0
	eher nein	0	0	0	0	0	0	0	0	0	0	0	0
	nein	0	0	0	0	0	0	0	0	0	0	0	0
gestaerkt	ja	4	2	2	5	2	5	3	3	7	6	8	8
	eher ja	1	2	3	1	4	2	5	5	1	3	2	0
	teils-teils	1	1	1	0	0	0	2	0	2	0	0	1
	eher nein	0	0	0	0	0	0	0	0	0	0	0	0
	nein	0	0	0	0	0	0	0	1	0	0	0	0
eigene_Bedürfnisse	ja	1	1	2	3	2	2	3	5	2	3	5	3
	eher ja	1	0	1	1	1	2	1	0	3	1	4	2
	teils-teils	2	2	3	1	3	1	2	2	5	3	1	2
	eher nein	0	0	0	0	0	2	2	0	0	0	0	1
	nein	2	1	0	1	0	0	2	2	0	2	0	1

	Antwortender Elternteil	Vater						Mutter					
	Zeitpunkt	MZP 1		MZP 2		MZP 3		MZP 1		MZP 2		MZP 3	
	Gruppe	120	90	120	90	120	90	120	90	120	90	120	90
Freunde_treffen	ja	1	2	1	3	3	4	0	1	0	1	3	3
	eher ja	0	1	0	1	0	1	2	1	1	2	0	3
	teils-teils	1	1	3	1	2	1	5	3	4	2	2	0
	eher nein	2	0	2	1	0	1	0	1	3	2	5	1
	nein	2	1	0	0	1	0	3	3	2	2	0	2
Arztbesuch_Behördengänge	ja	3	2	3	6	3	5	4	6	2	6	3	7
	eher ja	0	2	2	0	0	0	0	2	1	2	3	1
	teils-teils	1	0	0	0	2	1	3	0	3	1	1	1
	eher nein	0	1	1	0	0	0	0	0	0	0	0	0
	nein	2	0	0	0	1	1	3	0	4	0	3	0
partnerschaftlicheProbleme	ja	1	1	2	3	2	2	0	3	1	3	1	3
	eher ja	0	0	0	1	0	0	1	0	0	1	2	1
	teils-teils	0	1	0	0	0	1	2	3	3	1	2	1
	eher nein	0	0	0	0	1	1	1	1	0	0	1	0
	nein	5	3	4	2	3	3	6	2	6	4	4	4
Tagesablauf	ja	4	4	3	6	5	7	6	8	4	9	6	9
	eher ja	1	0	3	0	1	0	4	1	5	0	4	0
	teils-teils	1	1	0	0	0	0	0	0	1	0	0	0
	eher nein	0	0	0	0	0	0	0	0	0	0	0	0
	nein	0	0	0	0	0	0	0	0	0	0	0	0
Besonderheiten	ja	2	3	5	5	6	7	8	9	9	7	10	9
	eher ja	2	2	1	1	0	0	0	0	1	2	0	0
	teils-teils	0	0	0	0	0	0	0	0	0	0	0	0
	eher nein	0	0	0	0	0	0	0	0	0	0	0	0
	nein	2	0	0	0	0	0	2	0	0	0	0	0
Anregungen	ja	3	4	6	6	6	7	6	8	9	9	10	9
	eher ja	1	1	0	0	0	0	0	0	1	0	0	0
	teils-teils	2	0	0	0	0	0	4	1	0	0	0	0
	eher nein	0	0	0	0	0	0	0	0	0	0	0	0
	nein	0	0	0	0	0	0	0	0	0	0	0	0
freundlich	ja	6	5	6	6	6	7	10	8	10	9	10	9
	eher ja	0	0	0	0	0	0	0	1	0	0	0	0
	teils-teils	0	0	0	0	0	0	0	0	0	0	0	0
	eher nein	0	0	0	0	0	0	0	0	0	0	0	0
	nein	0	0	0	0	0	0	0	0	0	0	0	0

	Antwortender Elternteil	Vater						Mutter					
	Zeitpunkt	MZP 1		MZP 2		MZP 3		MZP 1		MZP 2		MZP 3	
	Gruppe	120	90	120	90	120	90	120	90	120	90	120	90
andere_Familienmitglieder	ja	2	3	3	4	6	7	3	5	3	5	4	6
	eher ja	0	1	3	1	0	0	1	2	2	2	3	1
	teils-teils	0	0	0	0	0	0	0	1	3	1	3	1
	eher nein	1	0	0	0	0	0	1	0	2	0	0	0
	nein	3	1	0	0	0	0	5	1	0	1	0	1
Info_Hilfsmöglichkeiten	ja	5	3	6	6	6	6	8	7	6	8	10	7
	eher ja	1	2	0	0	0	1	0	2	4	0	0	2
	teils-teils	0	0	0	0	0	0	0	0	0	0	0	0
	eher nein	0	0	0	0	0	0	0	0	0	0	0	0
	nein	0	0	0	0	0	0	2	0	0	1	0	0
pünktlich	ja	5	5	6	6	6	7	10	9	8	9	10	9
	eher ja	0	0	0	0	0	0	0	0	2	0	0	0
	teils-teils	1	0	0	0	0	0	0	0	0	0	0	0
	eher nein	0	0	0	0	0	0	0	0	0	0	0	0
	nein	0	0	0	0	0	0	0	0	0	0	0	0
Zusammenarbeit_Physio	ja	2	2	2	5	3	4	2	4	1	6	4	6
	eher ja	1	2	3	1	0	2	2	1	2	1	1	1
	teils-teils	1	0	0	0	1	0	3	1	4	0	0	0
	eher nein	0	0	0	0	0	0	1	0	1	1	1	1
	nein	2	0	0	0	1	0	2	2	2	0	3	0
Schlaf	ja	5	3	2	4	5	5	6	3	5	4	5	5
	eher ja	0	0	0	1	0	1	2	2	3	2	2	3
	teils-teils	1	1	4	1	1	1	2	2	1	2	3	1
	eher nein	0	0	0	0	0	0	0	2	0	1	0	0
	nein	0	1	0	0	0	0	0	0	1	0	0	0
Entwicklungsüberprüfung	ja	3	3	5	5	6	7	6	7	10	8	10	9
	eher ja	1	2	1	1	0	0	1	1	0	1	0	0
	teils-teils	0	0	0	0	0	0	0	0	0	0	0	0
	eher nein	0	0	0	0	0	0	1	0	0	0	0	0
	nein	2	0	0	0	0	0	2	0	0	0	0	0
Weiterempfehlen	ja	4	4	6	6	6	7	5	7	7	9	9	9
	eher ja	0	1	0	0	0	0	2	1	2	0	1	0
	teils-teils	0	0	0	0	0	0	1	0	1	0	0	0
	eher nein	0	0	0	0	0	0	0	0	0	0	0	0
	nein	2	0	0	0	0	0	2	1	0	0	0	0

PD Mag. Dr. Alexander Avian
+43-316-385-17873
alexander.avian@medunigraz.at

	Antwortender Elternteil	Vater						Mutter					
	Zeitpunkt	MZP 1		MZP 2		MZP 3		MZP 1		MZP 2		MZP 3	
	Gruppe	120	90	120	90	120	90	120	90	120	90	120	90
erneut_inAnspruchnehmen	ja	4	3	6	5	6	6	3	7	8	7	8	8
	eher ja	0	1	0	1	0	1	3	2	2	2	2	0
	teils-teils	0	0	0	0	0	0	1	0	0	0	0	1
	eher nein	0	0	0	0	0	0	1	0	0	0	0	0
	nein	2	1	0	0	0	0	2	0	0	0	0	0
Zeit_fürsich	ja	1	1	2	3	2	2	1	3	1	3	2	2
	eher ja	1	1	0	0	1	2	1	1	5	2	0	4
	teils-teils	1	1	3	3	2	1	3	3	1	3	3	2
	eher nein	1	1	1	0	0	2	2	2	1	1	4	1
	nein	2	1	0	0	1	0	3	0	2	0	1	0
Probleme_besprechen	ja	5	3	4	5	5	6	9	8	7	7	9	6
	eher ja	1	1	1	1	1	1	1	1	2	0	1	1
	teils-teils	0	1	1	0	0	0	0	0	0	0	0	1
	eher nein	0	0	0	0	0	0	0	0	1	1	0	0
	nein	0	0	0	0	0	0	0	0	0	1	0	1
gesund	ja	4	3	4	4	5	5	6	6	6	5	5	7
	eher ja	0	1	2	2	0	2	2	2	2	2	3	1
	teils-teils	2	1	0	0	1	0	2	1	2	2	2	1
	eher nein	0	0	0	0	0	0	0	0	0	0	0	0
	nein	0	0	0	0	0	0	0	0	0	0	0	0
Gesamtbeurteilung	sehr gut	2	4	5	6	6	7	6	8	9	8	10	8
	gut	2	1	1	0	0	0	2	1	1	1	0	1
	teils-teils	2	0	0	0	0	0	2	0	0	0	0	0
	schlecht	0	0	0	0	0	0	0	0	0	0	0	0
	sehr schlecht	0	0	0	0	0	0	0	0	0	0	0	0

PD Mag. Dr. Alexander Avian
+43-316-385-17873
alexander.avian@medunigraz.at

1.2 Antworten in drei Kategorien zusammengefasst

1.2.1 Häufigkeitstabelle der Antworten

	Antwortender Elternteil	Vater						Mutter					
	Zeitpunkt	MZP 1		MZP 2		MZP 3		MZP 1		MZP 2		MZP 3	
Gruppe		120	90	120	90	120	90	120	90	120	90	120	90
ernst_nehmen	ja / eher ja	6	5	6	6	6	7	10	9	10	9	10	9
	teils-teils	0	0	0	0	0	0	0	0	0	0	0	0
	nein / eher nein	0	0	0	0	0	0	0	0	0	0	0	0
Erklärungen_verstanden	ja / eher ja	6	5	6	6	6	7	10	9	10	9	10	9
	teils-teils	0	0	0	0	0	0	0	0	0	0	0	0
	nein / eher nein	0	0	0	0	0	0	0	0	0	0	0	0
Gespräche_zu_kurz	ja / eher ja	0	0	1	2	1	1	0	0	1	2	1	2
	teils-teils	0	2	0	0	1	0	0	2	1	1	2	1
	nein / eher nein	6	3	5	4	4	6	10	7	8	6	7	6
Gespräche_zu_selten	ja / eher ja	0	1	0	0	1	1	0	1	0	0	1	1
	teils-teils	0	0	0	0	0	0	0	0	1	0	1	0
	nein / eher nein	6	4	6	6	5	6	10	8	9	9	8	8
Wünsche	ja / eher ja	6	5	6	6	6	7	10	9	10	8	10	9
	teils-teils	0	0	0	0	0	0	0	0	0	1	0	0
	nein / eher nein	0	0	0	0	0	0	0	0	0	0	0	0
motiviert	ja / eher ja	6	5	6	6	6	7	10	9	10	9	10	9
	teils-teils	0	0	0	0	0	0	0	0	0	0	0	0
	nein / eher nein	0	0	0	0	0	0	0	0	0	0	0	0
Freuen	ja / eher ja	5	5	6	6	6	7	9	9	10	9	10	9
	teils-teils	1	0	0	0	0	0	1	0	0	0	0	0
	nein / eher nein	0	0	0	0	0	0	0	0	0	0	0	0
Sicherheit	ja / eher ja	5	4	6	6	6	7	8	7	9	9	10	9
	teils-teils	1	1	0	0	0	0	1	1	1	0	0	0
	nein / eher nein	0	0	0	0	0	0	1	1	0	0	0	0
Ängste_vermindern	ja / eher ja	4	4	2	6	2	6	7	7	6	9	9	9
	teils-teils	2	1	4	0	4	1	3	1	4	0	1	0
	nein / eher nein	0	0	0	0	0	0	0	0	0	0	0	0
gestaerkt	ja / eher ja	5	4	5	6	6	7	8	8	8	9	10	8
	teils-teils	1	1	1	0	0	0	2	0	2	0	0	1
	nein / eher nein	0	0	0	0	0	0	0	1	0	0	0	0
eigene_Bedürfnisse	ja / eher ja	2	1	3	4	3	4	4	5	5	4	9	5
	teils-teils	2	2	3	1	3	1	2	2	5	3	1	2

	nein / eher nein	2	1	0	1	0	2	4	2	0	2	0	2
Freunde_treffen	ja / eher ja	1	3	1	4	3	5	2	2	1	3	3	6
	teils-teils	1	1	3	1	2	1	5	3	4	2	2	0
	nein / eher nein	4	1	2	1	1	1	3	4	5	4	5	3
Arztbesuch_Behördengänge	ja / eher ja	3	4	5	6	3	5	4	8	3	8	6	8
	teils-teils	1	0	0	0	2	1	3	0	3	1	1	1
	nein / eher nein	2	1	1	0	1	1	3	0	4	0	3	0
partnerschaftlicheProbleme	ja / eher ja	1	1	2	4	2	2	1	3	1	4	3	4
	teils-teils	0	1	0	0	0	1	2	3	3	1	2	1
	nein / eher nein	5	3	4	2	4	4	7	3	6	4	5	4
Tagesablauf	ja / eher ja	5	4	6	6	6	7	10	9	9	9	10	9
	teils-teils	1	1	0	0	0	0	0	0	1	0	0	0
	nein / eher nein	0	0	0	0	0	0	0	0	0	0	0	0
Besonderheiten	ja / eher ja	4	5	6	6	6	7	8	9	10	9	10	9
	teils-teils	0	0	0	0	0	0	0	0	0	0	0	0
	nein / eher nein	2	0	0	0	0	0	2	0	0	0	0	0
Anregungen	ja / eher ja	4	5	6	6	6	7	6	8	10	9	10	9
	teils-teils	2	0	0	0	0	0	4	1	0	0	0	0
	nein / eher nein	0	0	0	0	0	0	0	0	0	0	0	0
freundlich	ja / eher ja	6	5	6	6	6	7	10	9	10	9	10	9
	teils-teils	0	0	0	0	0	0	0	0	0	0	0	0
	nein / eher nein	0	0	0	0	0	0	0	0	0	0	0	0
andere_Familienmitglieder	ja / eher ja	2	4	6	5	6	7	4	7	5	7	7	7
	teils-teils	0	0	0	0	0	0	0	1	3	1	3	1
	nein / eher nein	4	1	0	0	0	0	6	1	2	1	0	1
Info_Hilfsmöglichkeiten	ja / eher ja	6	5	6	6	6	7	8	9	10	8	10	9
	teils-teils	0	0	0	0	0	0	0	0	0	0	0	0
	nein / eher nein	0	0	0	0	0	0	2	0	0	1	0	0
pünktlich	ja / eher ja	5	5	6	6	6	7	10	9	10	9	10	9
	teils-teils	1	0	0	0	0	0	0	0	0	0	0	0
	nein / eher nein	0	0	0	0	0	0	0	0	0	0	0	0
Zusammenarbeit_Physio	ja / eher ja	3	4	5	6	3	6	4	5	3	7	5	7
	teils-teils	1	0	0	0	1	0	3	1	4	0	0	0
	nein / eher nein	2	0	0	0	1	0	3	2	3	1	4	1
Schlaf	ja / eher ja	5	3	2	5	5	6	8	5	8	6	7	8
	teils-teils	1	1	4	1	1	1	2	2	1	2	3	1
	nein / eher nein	0	1	0	0	0	0	0	2	1	1	0	0
Entwicklungsüberprüfung	ja / eher ja	4	5	6	6	6	7	7	8	10	9	10	9
	teils-teils	0	0	0	0	0	0	0	0	0	0	0	0

PD Mag. Dr. Alexander Avian
+43-316-385-17873
alexander.avian@medunigraz.at

	nein / eher nein	2	0	0	0	0	0	3	0	0	0	0	0
Weiterempfehlen	ja / eher ja	4	5	6	6	6	7	7	8	9	9	10	9
	teils-teils	0	0	0	0	0	0	1	0	1	0	0	0
	nein / eher nein	2	0	0	0	0	0	2	1	0	0	0	0
erneut_inAnspruchnehmen	ja / eher ja	4	4	6	6	6	7	6	9	10	9	10	8
	teils-teils	0	0	0	0	0	0	1	0	0	0	0	1
	nein / eher nein	2	1	0	0	0	0	3	0	0	0	0	0
Zeit_fürsich	ja / eher ja	2	2	2	3	3	4	2	4	6	5	2	6
	teils-teils	1	1	3	3	2	1	3	3	1	3	3	2
	nein / eher nein	3	2	1	0	1	2	5	2	3	1	5	1
Probleme_besprechen	ja / eher ja	6	4	5	6	6	7	10	9	9	7	10	7
	teils-teils	0	1	1	0	0	0	0	0	0	0	0	1
	nein / eher nein	0	0	0	0	0	0	0	0	1	2	0	1
gesund	ja / eher ja	4	4	6	6	5	7	8	8	8	7	8	8
	teils-teils	2	1	0	0	1	0	2	1	2	2	2	1
	nein / eher nein	0	0	0	0	0	0	0	0	0	0	0	0
Gesamtbeurteilung	Sehr gut / gut	4	5	6	6	6	7	8	9	10	9	10	9
	teils-teils	2	0	0	0	0	0	2	0	0	0	0	0
	schlecht / sehr schlecht	0	0	0	0	0	0	0	0	0	0	0	0

PD Mag. Dr. Alexander Avian
+43-316-385-17873
alexander.avian@medunigraz.at

1.2.2 Balkendiagramme der Häufigkeit der Antworten bei der letzten Befragung

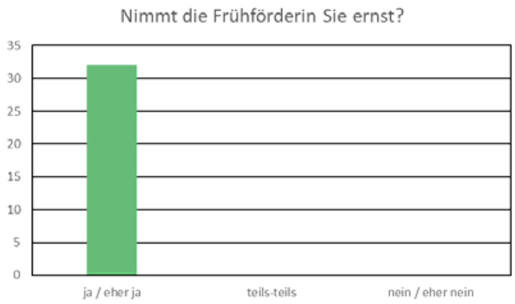

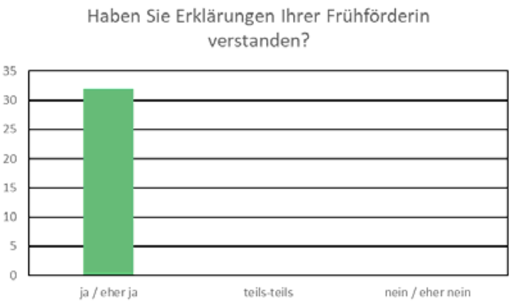

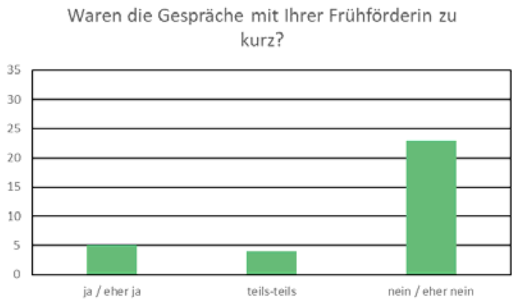

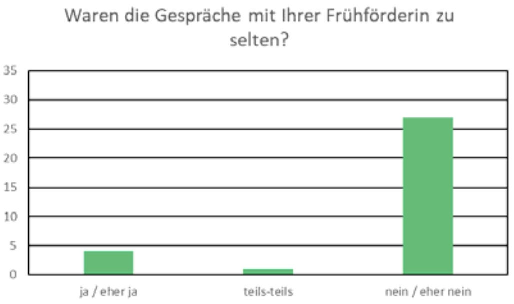

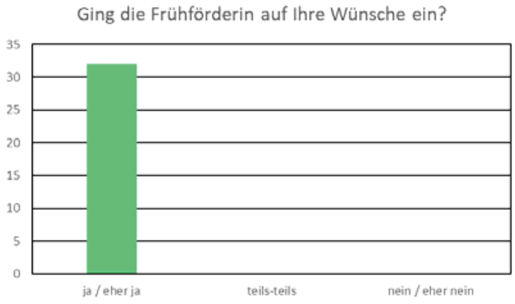

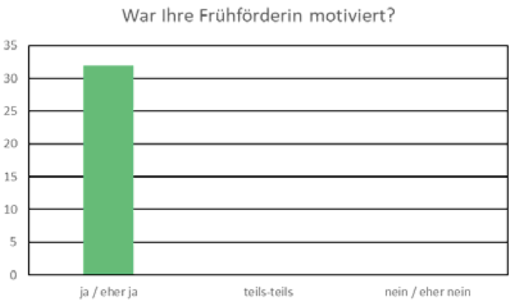

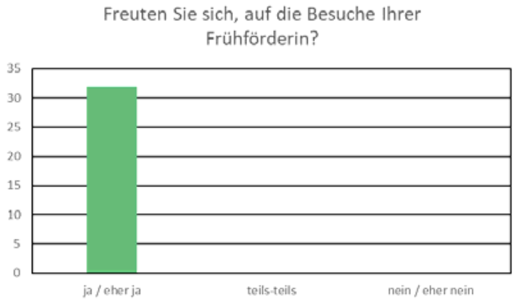

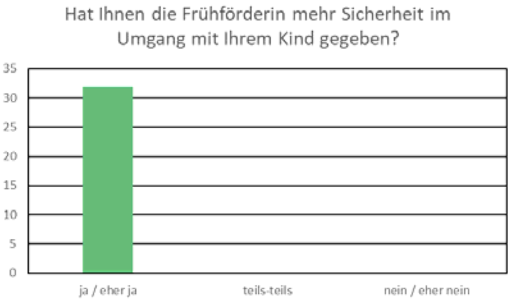

PD Mag. Dr. Alexander Avian
+43-316-385-17873
alexander.avian@medunigraz.at

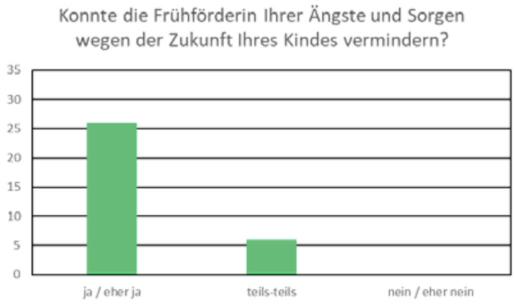

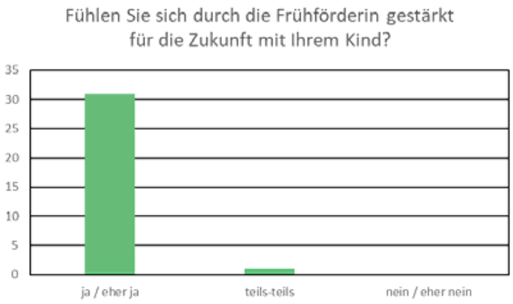

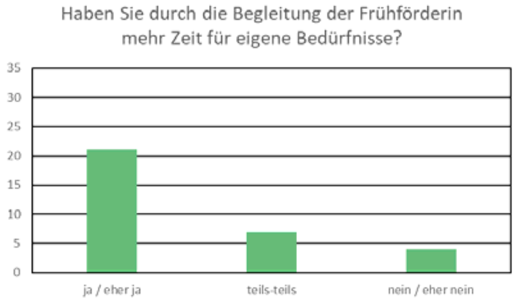

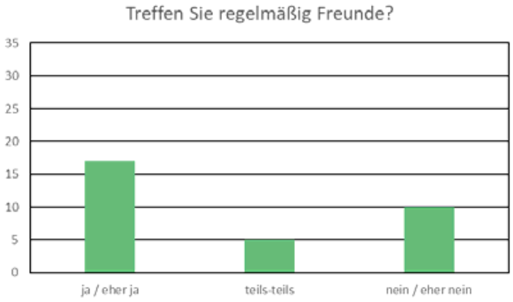

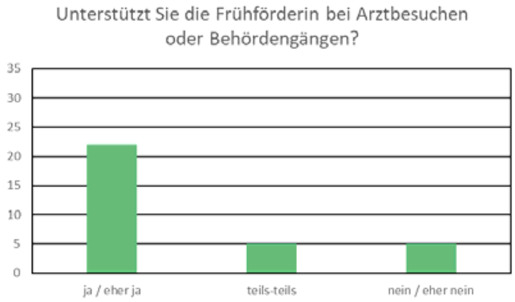

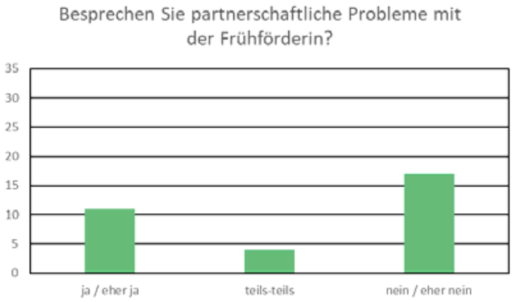

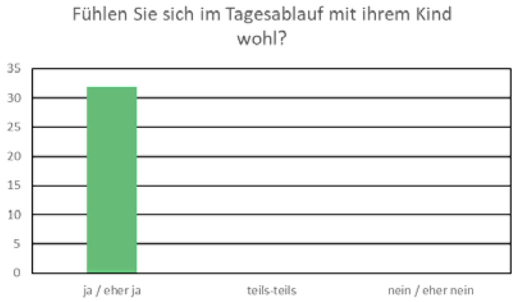

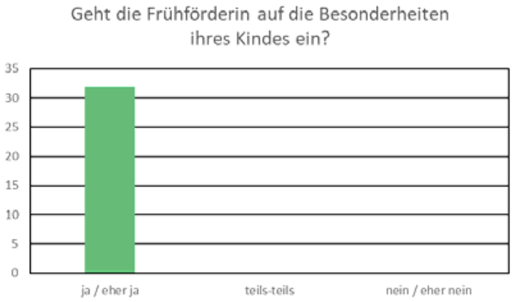

PD Mag. Dr. Alexander Avian
+43-316-385-17873
alexander.avian@medunigraz.at

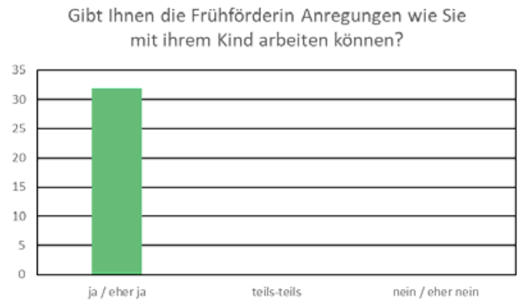
Gibt Ihnen die Frühförderin Anregungen wie Sie mit ihrem Kind arbeiten können?
35
30
25
20
15
10
5
0
ja / eher ja
teils-teils
nein / eher nein

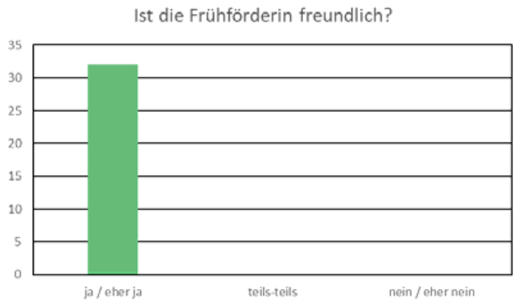
Ist die Frühförderin freundlich?
35
30
25
20
15
10
5
0
ja / eher ja
teils-teils
nein / eher nein

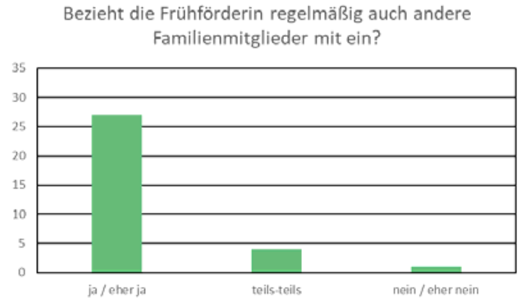
Bezieht die Frühförderin regelmäßig auch andere Familienmitglieder mit ein?
35
30
25
20
15
10
5
0
ja / eher ja
teils-teils
nein / eher nein

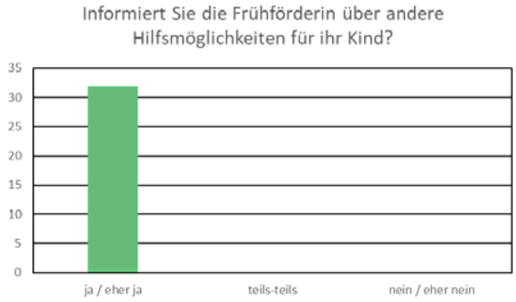
Informiert Sie die Frühförderin über andere Hilfsmöglichkeiten für ihr Kind?
35
30
25
20
15
10
5
0
ja / eher ja
teils-teils
nein / eher nein

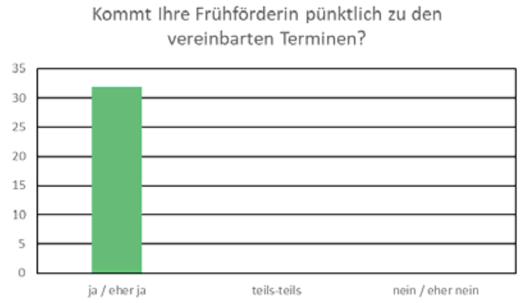
Kommt Ihre Frühförderin pünktlich zu den vereinbarten Terminen?
35
30
25
20
15
10
5
0
ja / eher ja
teils-teils
nein / eher nein

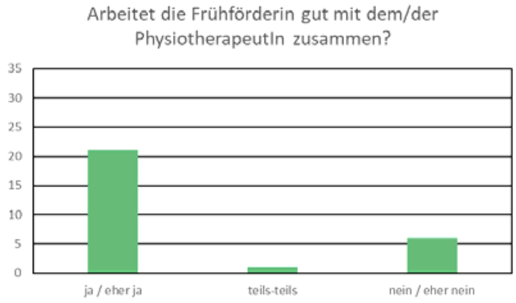
Arbeitet die Frühförderin gut mit dem/der PhysiotherapeutIn zusammen?
35
30
25
20
15
10
5
0
ja / eher ja
teils-teils
nein / eher nein

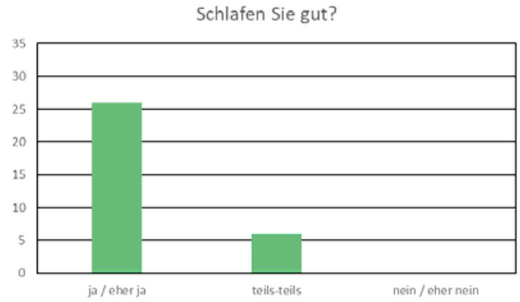
Schlafen Sie gut?
35
30
25
20
15
10
5
0
ja / eher ja
teils-teils
nein / eher nein

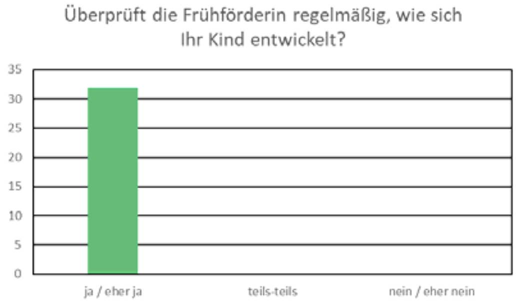
Überprüft die Frühförderin regelmäßig, wie sich Ihr Kind entwickelt?
35
30
25
20
15
10
5
0
ja / eher ja
teils-teils
nein / eher nein

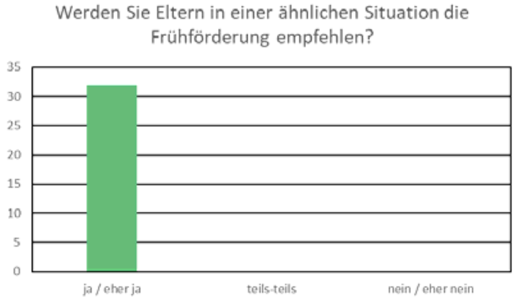

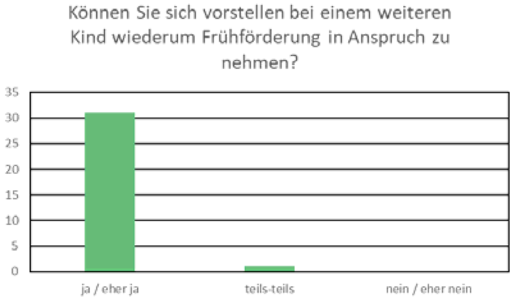

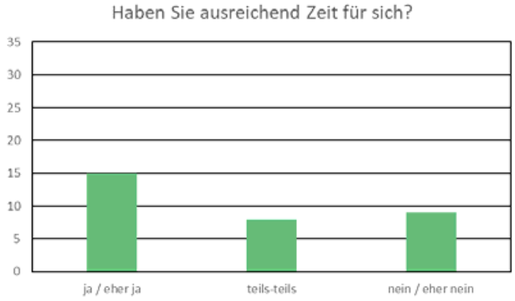

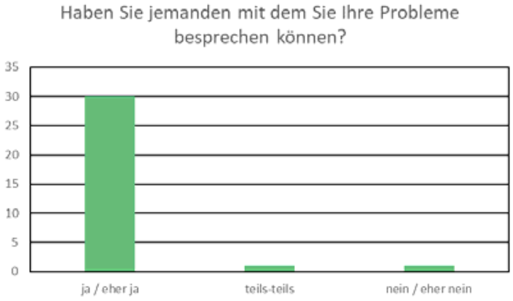

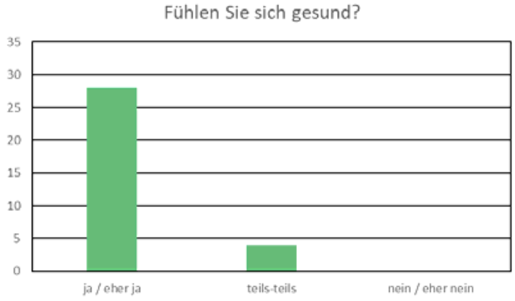

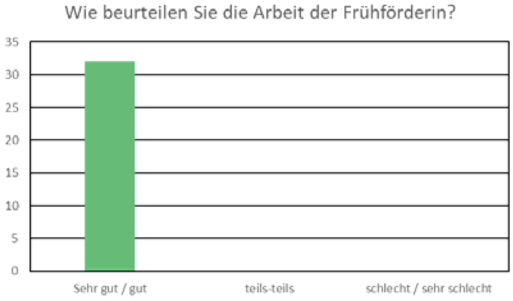

Institut für Medizinische Informatik, Statistik und Dokumentation
Medizinische Universität Graz
Auenbrugger Platz 2, 8036 Graz, Austria
PD Mag. Dr. Alexander Avian
+43-316-385-17873
alexander.avian@medunigraz.at
